# Commentary: Directions for Optimization of Photosynthetic Carbon Fixation: RuBisCO's Efficiency May Not Be So Constrained After All

**DOI:** 10.3389/fpls.2018.00929

**Published:** 2018-06-27

**Authors:** Guillaume G. Tcherkez, Camille Bathellier, Graham D. Farquhar, George H. Lorimer

**Affiliations:** ^1^Research School of Biology, Australian National University, ANU College of Science, Canberra, ACT, Australia; ^2^Department of Chemistry and Biochemistry, College of Computer, Mathematical and Natural Sciences, University of Maryland, College Park, MD, United States

**Keywords:** enzymatic activity, rubisco, isotope fractionation, enzyme kinetics and specificity, trade-offs

## Introduction

**Figure 1:**
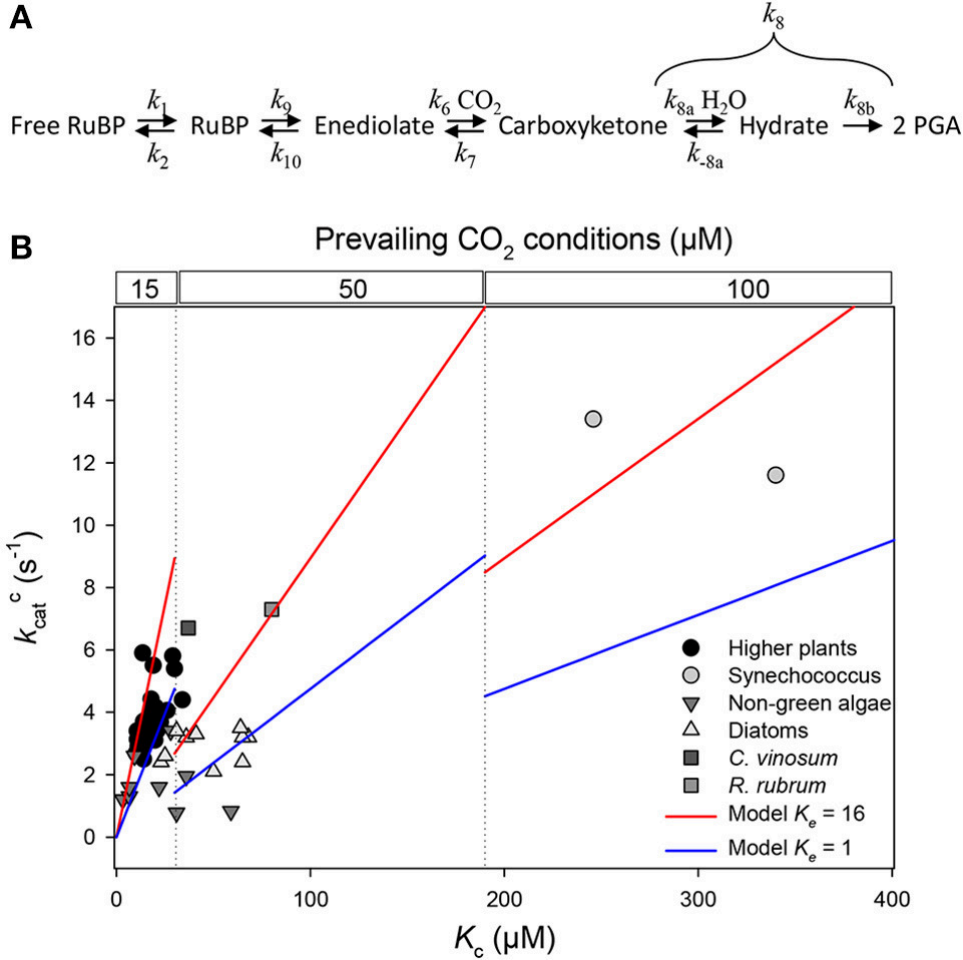
Relationship between Rubisco's turn-over rate for carboxylation (*k*${}_{\text{cat}}^{\text{c}}$) and the apparent Michaelis constant for CO_2_ (*K*_c_) using the same dataset tabulated by (Cummins et al., 2018). **(A)** Formal representation of the mechanism for carboxylation, with rate constants mentioned in main text. **(B)** Representation of *k*${}_{\text{cat}}^{\text{c}}$ against *K*_c_ using a linear scale on both axes. Steady-state kinetics are so that *k*${}_{\text{cat}}^{\text{c}}$ = *K*_c_·*k*_6_·*c*·*K*_e_/[(1 + *c*)·(1 + *K*_e_)] where *K*_e_ is enolization equilibrium constant *k*_9_/*k*_10_ and *c* is commitment to catalysis *k*_8_/*k*_7_ (Farquhar, 1979). It should be noted that *k*_6_ is not in s^−1^ but in μM^−1^ s^−1^ and thus not on the same scale for all organisms since it depends on prevailing subcellular CO_2_ concentration. The two linear models shown (blue and red lines) represent numerical examples of the relationship and assume that *k*_6_·[CO_2_] is fixed at 5 s^−1^ while *k*_6_ is subdivided into three domains of prevailing CO_2_ conditions varying with *K*_c_ (10, 50 and 100 μM). Calculations assume that enolization is efficient (*K*_e_ = 16, red) or poor (*K*_e_ = 1, blue) and that the commitment to catalysis is *c* = 95/5 = 19 (Lorimer et al., 1986).

Ribulose 1,5-bisphosphate carboxylase/oxygenase (Rubisco, EC 4.1.1.39) catalyzes the fixation of CO_2_ and O_2_ onto ribulose 1,5-bisphosphate (RuBP) during photosynthesis and photorespiration, respectively. This enzyme is required by nearly all photosynthetic organisms and its expression, structure, and mechanism have been intensively studied, with the ultimate objective of engineering a more “efficient” enzyme (i.e., faster and more specific to CO_2_). The reaction proceeds via a step-wise mechanism whereby RuBP is converted to an enediolate and then CO_2_ is added and the resulting 6-carbon (carboxyketone) intermediate is hydrated and cleaved (Figure 1A). Nevertheless, our current understanding of the chemical mechanism is limited and thus best ways to optimize the rate of CO_2_ fixation or affinity for CO_2_ are not totally clear. Therefore, comparisons of Rubisco kinetics from different organisms have been used to infer general rules that dictate variations in turn-over for carboxylation (*k*${}_{\text{cat}}^{\text{c}}$), apparent Michaelis constant for CO_2_ (*K*_c_), and specificity (*S*_c/o_). In their recent paper, Cummins et al. (2018) looked at published kinetic constants for a range of photosynthetic organisms and using linear regressions, concluded that “dissociation constants” for CO_2_ and O_2_ (rate constants for decarboxylation and deoxygenation) were relatively high and break the generally assumed relationship between *k*_cat_ and *S*_c/o_. Despite substantial variation in the chemical strategies of Rubiscos from different taxonomic groups that may exist, we believe that this analysis misinterprets implicit relationships between Rubisco rate constants, and overlooks experimental evidence (Table 1) for feeble rates of deoxygenation and decarboxylation.

**Table 1:** Direct evidence that the enolization equilibrium differs between Rubisco forms, and that decarboxylation and deoxygenation are negligible.

**Questions raised by Cummins et al. (2018)**	**Answer (yes/no)**	**Experimental evidence**	**References**
Is the decarboxylation rate of importance?	No	1. The partitioning (catalysis:decarboxylation) of the 6-carbon intermediate when it is fed to the enzyme has been shown to be at least 95:5.	Lorimer et al., 1986
		2. Should decarboxylation be substantial, we should observe a small ^12^C/^13^C kinetic isotope effect (^13^*V/K*) during carboxylation. In fact, ^13^*V/K* is given by α_eq_·(1 + *cα*_7_)/(1 + *c*) where α_eq_ is the equilibrium isotope effect of carboxylation, α_7_ is the kinetic isotope effect of decarboxylation and *c* the commitment to catalysis (*c* = *k*_8b_/*k*_7_). CO_2_ addition on sugars forming a ternary C atom favors ^13^C by about 3%0 so that α_eq_ is about 0.997. A value of *c* = 1 gives a fractionation within 0.997-1.011 for possible values of α_7_ between 1 and 1.030 (feasible range for a ^12^C/^13^C kinetic isotope effect). It is therefore impossible to match the observed isotope effect in most organisms (^13^*V/K* ≈ 1.030 in higher plants) unless assuming extremely high values of the isotope effect for decarboxylation (about 1.070).	O'Leary and Yapp, 1978; Roeske and O'Leary, 1984, 1985; Rishavy and Cleland, 1999
Is enolization variable and thus can *K*_R_ (and γ_c_) change a lot between Rubiscos?	Yes	1. A typical example is *Rhodospirillum rubrum*, which does not fit the empirical linearization used by Cummins *et al*. (2018). In fact, the intrinsic ^1^H/^2^H isotope effect (RuBP deuterated in H3) on maximal velocity (^D^*V*) when enolization becomes rate-limiting (at low pH) is clearly lower in *R. rubrum* than in spinach; in addition, the isotope effect at limiting RuBP (^D^*V/K*) is unity in *R. rubrum* but increases at low pH, contrary to what is observed in spinach. The enzyme of *R. rubrum* can also easily exchange the H3 proton with the solvent.	Saver and Knowles, 1982; Van Dyk and Schloss, 1986
		2. There are considerable differences in the ability to carboxylate xylulose-1,5-bisphosphate (C3 stereoisomer of RuBP) between higher plants, prokaryotes and red algae, showing that the mechanistic constraints on H3 abstraction and thus stereochemistry of enolization differ between Rubisco forms.	Pearce, 2006
Is the deoxygenation rate of importance?	No	1. O_2_ addition forms a peroxide. In general, oxygenation to a peroxide is irreversible and thus deoxygenation of a peroxide is extremely unlikely.	Frankvoort, 1978; Lorimer, 1981
		2. Should the peroxide be deoxygenated, deoxygenation would not be the reverse of oxygenation because the spin-forbidden character of oxygenation requires excited chemical forms that are unlikely to be reformed. In practice, going backwards from the peroxide to the enediolate is strongly thermodynamically disfavored.	Jonsson, 1996; Bathellier et al., 2018
		3. As with ^13^C (above), the ^16^O/^18^O isotope effect during oxygenation (^18^*V/K* ≈ 1.021) indicates that an important commitment to deoxygenation is not credible.	Guy et al., 1993

## Simple linear regression is unlikely to be representative

It is common practice to use linear relationships between kinetic parameters in order to facilitate our understanding of the implicit linkage between rate constants of the mechanism. However, this technique is difficult to apply to Rubisco kinetics because no combination of experimentally accessible kinetic parameters (*k*${}_{\text{cat}}^{\text{c}}$, *K*_c_, *S*_c/o_) gives access to individual rate constants. Basically, Cummins et al. (2018) use the relationship *K*_c_ = (*k*${}_{\text{cat}}^{\text{c}}$ + γ_c_*k*_7_)/*K*_R_*k*_6_ (where *k*_6_ and *k*_7_ are the rate constants associated with CO_2_ addition [carboxylation *per se*] and decarboxylation, respectively^1^; γ_c_ is a complex parameter that integrates rate constants of enolization as well as hydration and cleavage) in order to find 1/*K*_R_*k*_6_ and γ_c_*k*_7_/*K*_R_*k*_6_ by linear regression across enzymes from a variety of photosynthetic organisms. As they recognize themselves, there is no linear relationship between *K*_c_ and *k*${}_{\text{cat}}^{\text{c}}$ (replotted in Figure 1B). Therefore, they used either (i) a selection of points (typically, one taxonomic group) to minimize non-linearity or (ii) used a log-transformation with subsequent re-linearization by Taylor expansion. The first method gives a considerable range of values between taxonomic groups (negative or positive slope), and the second method disregards conditions of validity to perform a Taylor expansion (i.e. to neglect second-order terms). Computed coefficients 1/*K*_R_*k*_6_ and γ_c_*k*_7_/*K*_R_*k*_6_ are in fact very unlikely to be constant because: (i) *K*_R_ directly depends on RuBP enolization equilibrium constant (*K*_e_) since *K*_R_ = *K*_e_/(1 + *K*_e_), which varies between Rubisco forms (Table 1); (ii) the rate constant for carboxylation (*k*_6_) and/or decarboxylation (*k*_7_) may vary between Rubisco forms; and (iii) γ_c_ comprises rate constants of enolization as well as hydration and cleavage.

There is experimental evidence that hydration is a very efficient process, that is, the on-enzyme hydration equilibrium of the carboxyketone substantially favors the hydrated form (Lorimer et al., 1986). Furthermore, (stereo)chemical constraints on the mechanism indicate that CO_2_ addition and hydration may be concerted (Cleland et al., 1998). Mathematically, this means that γ_c_ must be a relatively small number, close to *k*${}_{\text{cat}}^{\text{c}}$/*k*_8a_ where *k*_8a_ denotes the rate constant associated with hydration [denoted as *k*_7_ in Cummins et al. (2018)]. Also, *k*${}_{\text{cat}}^{\text{c}}$ can be rearranged to *k*_9_*k*_8b_/(*k*_9_ + *k*_8b_), making apparent the rate constant of enolization (*k*_9_). There is also direct evidence that the enolization equilibrium varies between Rubisco forms, and this probably contributes to explaining the non-linearity of the *k*${}_{\text{cat}}^{\text{c}}$/*K*_c_ relationship, as explained in Table 1 and (Tcherkez, 2013). In other words, the commitment to, and the transition state involved in enolization differ significantly between Rubisco forms such that the enolization equilibrium is an important variable in the landscape of Rubisco kinetic parameters, in addition to carboxylation (*k*_6_) and processing (*k*_8_).

## Decarboxylation and deoxygenation are negligible in wild-type rubisco

Linear regressions carried out by Cummins et al. (2018) provide an estimate of γ_c_*k*_7_ (the product of γ_c_ and the decarboxylation rate constant, *k*_7_) which is found to be of the same order of magnitude (3–4 s^−1^) as *k*${}_{\text{cat}}^{\text{c}}$ itself, meaning a low commitment of the enzyme to catalysis (*k*${}_{\text{cat}}^{\text{c}}$/γ_c_*k*_7_ ≈ 1). Such a high decarboxylation rate clearly contradicts experimental evidence (Table 1). We nevertheless recognize that mutant Rubisco forms can be impacted on decarboxylation, as we previously assumed in the L335V mutant to explain the typically low ^12^C/^13^C isotope effect on *V/K* (McNevin et al., 2007). Kinetic fitting of Rubisco velocity carried out by McNevin et al. (2006) suggested modest-to-high values of decarboxylation but these authors explicitly mentioned that computations were unable to give a reliable value, with no improvement of residuals whatever *k*_7_ may be. Deoxygenation is even less likely than decarboxylation for fundamental reasons summarized in Table 1.

## Kinetic parameters are constrained by both chemistry and environment

Taken as a whole, while we recognize that the attempt of Cummins et al. (2018) is valuable in trying to extract implicit rate constants from readily observable kinetic parameters, we believe that concluding that decarboxylation and deoxygenation are quantitatively important is not plausible. Our assumption published in Tcherkez et al. (2006) that Rubisco's evolutionary strategy involves complementarity of the active site to the transition-state, referred to as “tight-binding hypothesis” by Cummins et al. (2018), does not necessarily include a preferential change in the rate constant for carboxylation (*k*_6_) instead of *k*_7_ (decarboxylation), contrary to their claim. Rubisco adaptive value integrates not only catalytic “efficiency” (*k*${}_{\text{cat}}^{\text{c}}$/*K*_c_) but also specificity (*S*_c/o_), in the prevailing environmental CO_2_/O_2_ conditions. Even in diatoms which show variation in *K*_c_ while having a rather constant *k*${}_{\text{cat}}^{\text{c}}$ (Young et al., 2016), there is a relationship with CCM protein abundance and composition (such as the occurrence of carbonic anhydrase isoform δ) and thus subcellular CO_2_ concentrations (Young and Hopkinson, 2017) (see also Figure 1B). Also, it should be kept in mind that some residues of the active site are involved in several steps, such as *R. rubrum* Lys 166 which is involved in both enolization and hydration + cleavage, providing a chemical basis for the interdependence of kinetic parameters (Harpel et al., 2002). Therefore, the analysis described in Cummins et al. (2018) does not provide evidence that Rubisco kinetics are “not so constrained.”

## Author contributions

All authors listed have made a substantial, direct and intellectual contribution to the work, and approved it for publication.

### Conflict of interest statement

The authors declare that the research was conducted in the absence of any commercial or financial relationships that could be construed as a potential conflict of interest.

## Footnotes

^1^Here, we use the original rate constant numbering used to describe Rubisco kinetics in Farquhar (1979).
